# Carotid Intima Media Thickness in Nondiabetic Hypertensive Nigerians: Role of Fasting and Postprandial Blood Glucose

**DOI:** 10.1155/2016/1429451

**Published:** 2016-04-10

**Authors:** B. N. Okeahialam, S. A. Muoneme, H. O. Kolade-Yunusa

**Affiliations:** ^1^Department of Medicine, Jos University Teaching Hospital, Jos 930001, Nigeria; ^2^Department of Radiology, Jos University Teaching Hospital, Jos 930001, Nigeria

## Abstract

*Background/Aims*. Carotid intima media thickness (CIMT) tracks atherosclerotic vascular disease. Hypertension and diabetes chiefly contribute to atherosclerosis with 75% of symptomatic cardiovascular disease cases having dysglycaemia even in normal cases. Hypothesising that postprandial hyperglycaemia contributes to cardiovascular morbidity, we sought to determine if any relationship existed between glycaemic profile in nondiabetic hypertensives and atherosclerosis.* Methods*. In a study of CIMT in nondiabetic, statin-naïve hypertensives, we evaluated fasting blood glucose (FBG) and 2-hour postprandial sugar (2hPPBG) in the patients and compared them with the CIMT. CIMT was measured on both sides, 1 cm proximal to the carotid bulb using a 7.5 mHz transducer of ALOKA SSD-3500 ultrasound machine.* Results*. The subjects with complete data were 86 (63 F). The mean (SD) of CIMT was 0.89 (0.15) mm, FBG 4.8 (0.097) mmol/L, and 2hPPBG 6.5 (1.81) mmol/L. There was no significant correlation between FBG and 2hPPBG with CIMT. Blood pressure had no bearing on this. When blood glucose data were divided into quartiles and post hoc multiple comparison was done, there was significant difference in CIMT for the different ranges. This was not so for 2hPPBG.* Conclusion*. Though expected from other studies, we did not show any significant correlation between FBG and 2hPPBG status and CIMT. This may be our pattern as the degree of excursion of 2hPPBG was low. There may be a threshold level above which PPBG starts to impact CIMT.

## 1. Introduction

Carotid intima media thickness (CIMT) has been shown to have a relationship with atherosclerotic vascular events in various vascular beds [1]. As a result, though values differ by race, it is a robust measure of subclinical development of atherosclerosis [2]. Several risk factors contribute to atherosclerosis with hypertension and diabetes as chief contributors [3]. Accumulating evidence suggests that about 75% of symptomatic cardiovascular disease patients have dysglycaemia [4]. In a significant number, this is not detected by increase in fasting or postprandial blood sugar; the latter is even seen to be of greater impact [5]. In fact, postprandial hyperglycaemia but not fasting hyperglycaemia has been shown to independently predict the occurrence of cardiovascular events [6]. Even in patients with normal glucose metabolism, postload glycaemia correlates with the risk of cardiovascular and noncardiovascular deaths [7].

In an earlier study in our centre, we showed that fasting blood glucose (FBG) and 2-hour postprandial blood glucose (2hPPBG) contribute significantly to cardiovascular disease burden in Nigerian Africans with diabetes mellitus [8]. With the hyperglycaemic spikes of postprandial hyperglycaemia inducing oxidative stress that leads to endothelial dysfunction [9], a putative mechanism for atherosclerosis resulting from hyperglycaemia was established. With the hypothesis that postprandial blood sugar even in normoglycaemic patients contributes to cardiovascular morbidity, we decided to see if glycaemic profile of nondiabetic hypertensives plays a significant role in atherosclerosis. No such study to our knowledge has been carried out in Nigeria.

## 2. Materials and Methods

As part of a study in our centre that looked at CIMT in nondiabetic (history and laboratory confirmed) statin-naïve hypertensives, we evaluated 2hPPBG in the patients who underwent the CIMT study between November 2012 and February 2013. The study was approved by the Ethics and Research Committee of Jos University Teaching Hospital. The patients who were hypertensives recruited from the Cardiology Clinic were enrolled if they had no history of diabetes and use of statin or antidiabetic therapy and have normal FBG. After the CIMT evaluation by H. O. Kolade-Yunusa, whose values the physicians (B. N. Okeahialam and S. A. Muoneme) were blind to, they underwent 2hPPBG assay in the Diabetes Clinic blood glucose side laboratory of the hospital in the Medical Outpatient Department (MOPD). They were advised to go early to the MOPD fasting but with what they would usually eat for breakfast. There was no attempt to make the meal uniform so that their individual glucose profiles would not be altered. They were then asked to eat and report 2 hours afterwards for the test. The thumb of the hand was cleaned with methylated spirit and allowed to dry and then pricked with a lancet. A drop of capillary was put on the strip of SD Codefree Blood Glucose monitoring system (SD Biosensor) made in Republic of Korea by Suwon-Si Gyeonggi-do 443-813 for MT Promedt Consulting GmbH, St. Ingbert, Germany. Usually tests done in this side laboratory are quality controlled by comparing with laboratory venous blood glucose tests at regular intervals.

The examination of the CIMT was done in the Radiology Department of the hospital using the 7.5 mHz linear transducer with an ALOKA SSD-3500 ultrasound scanner equipped Doppler facility. Patients were requested to remove jewelry around the neck. A B mode ultrasound of the right and left common carotid arteries (CCA) was acquired with patients lying supine with a pillow support under the neck. This was to achieve the desired neck extension, and the head was turned 45 degrees away from the side being scanned. Three measurements were taken on both sides in the longitudinal plane at the point of maximal thickness on the far wall of both CCA, 1 cm proximal to the carotid bulb where it is clear of plaques. The carotid bulb was defined as the point where the far wall deviated away from the parallel plane of the distal CCA. The intima medial thickness was measured as the distance between the inner echogenic line representing the intima-blood interface and the outer echogenic line representing the adventitia-media junction. The mean of 3 measurements on each side was recorded for that side.

### 2.1. Statistics

The data set, expressed as mean (SD), was analysed using SPSS 17.0. Analysis of variance, Student's *t*-test, and post hoc multiple comparisons (with the Bonferroni correction) were used as appropriate to determine the degree of difference or association. Blood glucose data were categorized into quartiles to ensure nearly equal numbers and correlated with CIMT looking for any trend. A *p* value greater than or equal to 0.05 was considered statistically significant.

## 3. Results

A total of 86 subjects (63 F, 23 M) had complete blood sugar and CIMT data. Their ages ranged from 29 to 72 years with a mean (SD) of 47.47 (10.49). Treatment of their blood pressures varied depending on their cardiovascular profiles. They were however statin naïve. Blood pressure was controlled (less than 140/90 mmHg) in 70.2% and uncontrolled (greater than or equal to 140/90 mmHg) in 29.8%. For CIMT the mean of the values for right and left CIMT was used. The mean (SD) FBG was 4.8 (0.097) mmol/L; range was 1.9 to 6.6 mmol/L, which is in consonance with their nondiabetic status. The mean (SD) 2hPPBG was 6.5 (1.81) mmol/L; range was 3.9 to 15.8 mmol/L. The mean (SD) CIMT was 0.89 (0.15). Group comparison of continuous data (FBG, 2hPPBG, and CIMT) was performed using ANOVA. There was no significant correlation between FBG and CIMT (*p* = 0.324) as well as 2hPPBG and CIMT (*p* = 0.472).

Wondering whether control status of the hypertensives at the point of CIMT assessment impacted on their CIMT values, we dichotomized patients based on control status (control, BP < 140/90 mmHg; no control, BP ≥ 140/90 mmHg). It turned out that the *t*-test for the CIMT difference by group was not significant (*p* = 0.229).

With CIMT not significantly correlating with FBG and 2hPPBG, the data were divided into quartiles. The CIMT difference between groups for FBG tended to but did not attain statistical significance (*p* = 0.09). On post hoc multiple comparison, there was significance in CIMT at *p* = 0.05 level for the different ranges (≤4.10 mmol/L, 4.11–4.80 mmol/L, 4.81–5.50 mmol/L, and ≥5.51 mmol/L). See Table 1.

The difference between groups for 2hPPBG was also not statistically significant (*p* = 0.57). On post hoc multiple comparison, there was also no statistically significant difference, though CIMT tended to increase as 2hPPBG rose (≤5.30 mmol/L, 5.31–6.20 mmol/L, 6.21–7.20 mmol/L, and ≥7.21 mmol/L). See Table 2.

## 4. Discussion

In this work, we have shown that, in nondiabetic hypertensives, both FBG and 2hPPBG have no significant bearing on CIMT. This is a bit surprising given the position that, even in nondiabetic individuals, blood glucose levels constitute a morbi-mortality risk [10]. There are other studies with contrary findings suggesting that there is a relationship between markers of nonfatal cardiovascular diseases (CVD) like IMT [11] and coronary artery calcium [12] and markers of hyperglycaemia in individuals without diabetes mellitus (DM). However, like our study, van't Riet et al. [13] did not register any significant association between FBG and 2hPPBG (except for HbA1C) with nonfatal CVD clinical outcome measures.

This may well be the pattern in our environment. In an earlier report [14] we showed that FBG and 2hPPBG in nondiabetic hypertensives were in the same range as nondiabetic, nonhypertensive controls, in contrast to a sex and age matched cohort of diabetic nonhypertensives. If blood sugars in nondiabetic hypertensives are similar to nondiabetic nonhypertensives, then glycaemic profile in the former should add no CVD risk.

The lack of significant association of glycaemic profile in this nondiabetic hypertensive population with CIMT, a measure of subclinical CVD, may have to do with low degree of excursions of the postprandial blood sugar or a threshold effect. In this study only 1 out of 86 had an abnormal 2hPPBG of 15.8 mmol/L. The mean value of the 2hPPBG in this study is normal, 6.5 mmol/L with a narrow standard deviation of 1.81 mmol/L. They could not as a result be said to have chronic hyperglycaemia which induces glucose toxicity of the vascular endothelial cells [15]. This toxicity is mediated by oxidative stress independent of other CVD risk factors [16]. The result of this oxidative stress is impairment of nitric oxide (NO) mediated endothelial function. This manifests in reduced flow-mediated vasodilatation in patients with impaired glucose tolerance (IGT) compared to normal subjects [5]. In our present study, only 14/86 (16,3%) had IGT defined as 2hPPBG ≥7.8 mmol/L [17]. With the minority having IGT, this endothelial dysfunction contribution will be minimal. Increases in postprandial glucose acutely lower flow-mediated vasodilatation in healthy subjects as well as those with IGT and frank diabetes mellitus, with the latter groups showing greater reduction [9].

When the 2 sugar profiles FBG and 2hPPBG were distributed into quartiles, an interesting feature emerged. The CIMT mean between groups tended to, but did not attain, statistical significance. For 2hPPBG, there was no such tendency to difference in CIMT. On post hoc multiple comparison, there was significant difference in CIMT between the 4 groups, with CIMT rising as FBG rose. For 2hPPBG, post hoc multiple comparison showed no significant difference. The degree of rise in CIMT as 2hPPBG rose was less than for FBG. It would therefore appear that once the patient is not diabetic and FBG normal, postprandial blood glucose does not rise to levels where they are toxic to vascular endothelium and induce NO related dysfunction. However, the higher the FBG, albeit normal and not qualifying for diagnosis of DM, the higher the CIMT. This gets worse for the impaired fasting glycaemia group (defined as FBG ≥ 5.6 mmol/L) and agrees with the suggestion that a continuous relationship exists between increasing glucose levels and increasing endothelial dysfunction [10]. Nasir et al. [18] in Brazil also documented that FBG in the upper normal range is associated with subclinical CVD and the presence of increased coronary artery calcium burden.

This study may face a limitation from the known CIMT measurement errors that occur in relation to variables which acutely alter vascular tone, namely, exercise, drugs, foods and beverages, and diurnal rhythm [19]. Also the sample size is not optimal and limits the statistical power of the study. It would have been better if the 2hPPBG was done on the same day and all in the Chemical Pathology laboratory on whole venous blood. However though slight difference exists, laboratory venous blood glucose assay correlates significantly with capillary blood sugar tests [20].

To conclude, in nondiabetic hypertensives, risk of atherosclerosis is higher in the impaired fasting glycaemia group. Apart from controlling the blood pressure, life-style measures to reduce blood sugar stand to benefit them from that perspective. For them 2hPPBG excursion does not appear to put them at any significant additional risk for atherosclerosis. It would make an interesting study to see how much additional atherosclerotic burden postprandial blood sugar puts on diabetic patients albeit controlled.

## Figures and Tables

**Table 1:** CIMT relationship with FBG by quartile levels.

FBG (mmol/L)	Number	Mean CIMT (SD)
≤4.10	23	0.86 (0.11) mm
4.11–4.80	23	0.92 (0.16) mm
4.81–5.50	19	0.83 (0.12) mm
≥5.51	21	0.93 (0.19) mm

*F* value for difference between groups = 2.21 (*p* = 0.09).

**Table 2:** CIMT relationship with 2hPPBG by quartile levels.

2hPPBG (mmol/L)	Number	Mean CIMT (SD)
≤5.30	23	0.86 (0.11) mm
5.31–6.20	21	0.87 (0.16) mm
6.21–7.20	24	0.91 (0.19) mm
≥7.21	18	0.91 (0.19) mm

*F* value for difference between groups = 0.67 (*p* = 0.57).
